# Association of Genetic Variants With Body-Mass Index and Blood Pressure in Adolescents: A Replication Study

**DOI:** 10.3389/fgene.2021.690335

**Published:** 2021-09-01

**Authors:** Danick Goulet, Jennifer O’Loughlin, Marie-Pierre Sylvestre

**Affiliations:** ^1^École de santé publique, Université de Montréal, Montréal, QC, Canada; ^2^Centre de Recherche du Centre Hospitalier de l’Université de Montréal (CRCHUM), Montréal, QC, Canada

**Keywords:** adiposity, blood pressure, genetic association study, adolescence, replication

## Abstract

The strong correlation between adiposity and blood pressure (BP) might be explained in part by shared genetic risk factors. A recent study identified three nucleotide variants [rs16933812 (*PAX5*), rs7638110 (*MRPS22*), and rs9930333 (*FTO*)] associated with both body mass index (BMI) and systolic blood pressure (SBP) in adolescents age 12–18years. We attempted to replicate these findings in a sample of adolescents of similar age. A total of 713 adolescents were genotyped and had anthropometric indicators and blood pressure measured at age 13, 15, 17, and 24years. Using linear mixed models, we assessed associations of these variants with BMI and SBP. In our data, rs9930333 (*FTO*) was associated with body mass index, but not systolic blood pressure. Neither rs16933812 (*PAX5*) nor rs7638110 (*MRPS22*) were associated with body mass index or systolic blood pressure. Although, differences in phenotypic definitions and in genetic architecture across populations may explain some of the discrepancy across studies, nucleotide variant selection in the initial study may have led to false-positive results that could not be replicated.

## Introduction

In 2015, high blood pressure (BP) and high body mass index (BMI) were the first and fourth risk factors, respectively, for disability-adjusted life-years (Forouzanfar et al., 2016). BP and adiposity are strongly correlated (Man et al., 2020), and higher adiposity is an important risk factor for elevated BP, both in adults (Jayedi et al., 2018) and adolescents (Chorin et al., 2015). The common variants/multiple disease hypothesis first introduced by Becker (2004) posits that correlation between related traits such as adiposity and BP, may be explained at least in part by shared genetic risk factors. The estimated genetic correlation between 24h ambulatory systolic BP (SBP) and BMI (r_g_: 0.33; Man et al., 2020) suggests the presence of common genes affecting both traits, but further investigation is needed to identify pleiotropic genes at play.

Melka et al. (2012) identified three single nucleotide polymorphisms (SNPs; rs16933812 on *PAX5*, rs7638110 on *MRPS22* and rs9930333 on *FTO*) associated with both BMI and SBP in a population-based sample of 598 adolescents age 12–18years in Saguenay-Lac-St-Jean, Quebec, Canada as part of the Saguenay Youth Study (SYS). However, these results have not yet been replicated in an independent sample. Variants rs16933812 (*PAX5*) and rs7638110 (*MRPS22*) were newly discovered variants in the SYS but they were not associated with either BP or BMI in recent meta-analyses for these traits (Evangelou et al., 2018; Yengo et al., 2018). *FTO* is a gene well-known for its association with obesity phenotypes and rs9930333 (*FTO*) has been associated with BMI in recent adult (Yengo et al., 2018) and child (Vogelezang et al., 2020) meta-analyses, but not with BP (Evangelou et al., 2018).

There is evidence that some BP-associated variants are expressed differently in youth than in adults, highlighting the need for replication of newly discovered findings, specifically in adolescents. A large genome-wide association study (GWAS) reported 31 genetic variants that had age-specific effects on adult BP, one of which had opposite effects in younger vs. older persons (Simino et al., 2014). Some suggest the emergence of novel genetic effects during adolescence, the activation of which is probably related to pubertal changes (Kupper et al., 2006). Others have noted that not all genetic variants identified in adult samples are associated with BP in children (Wang et al., 2015), suggesting that genetic variants have an age-dependent effect on BP. However, genetic association studies for BMI and BP in youth compared to adults are scarce (Albuquerque et al., 2015; Ahn and Gupta, 2018) and rarely replicated.

In the current study, we sought to replicate main findings of Melka et al. (2012) that SNPs on three genes (rs16933812 on *PAX5*, rs7638110 on *MRPS22*, and rs9930333 on *FTO*) are associated with both BMI and SBP. We used data from the Nicotine Dependence In Teens (NDIT) study (O’Loughlin et al., 2014), a longitudinal investigation of adolescents in Montreal, Quebec, Canada followed from age 12 to 24 (i.e., from 1999 to 2012). The NDIT sample was similar in age and geographic location to SYS.

## Materials and Methods

Data were drawn from the NDIT study, a longitudinal investigation of the natural course of nicotine dependence across adolescence (O’Loughlin et al., 2014). A total of 1,294 grade 7 students age 12–13years were recruited in 1999–2000 in 10 Montreal-area (Canada) high schools purposively selected to include French and English schools, schools located in rural, urban, and suburban areas, and schools serving socioeconomically diverse neighborhoods. Data was collected in self-administered questionnaires across 23 data collection cycles. Data in cycles 1–20 were collected in grade 7–11 (i.e., the last year of high school in the province of Quebec) every 3months during the 10-month school year over 5years. Post high school data were collected in cycles 21, 22, and 23 when participants were age 20, 24, and 31years on average. A detailed description of study variables collected in self-administered questionnaires including the cycles in which the variable was available, the questionnaire item(s), response choices, whether the questionnaire items used to measure specific variables changed over time and recoding for analysis is available in Supplementary Table 1.

DNA samples were collected from 943 of 1,294 NDIT participants (73%). The 713 participants who were unrelated, of European descent, had at least one BMI or SBP measure available, and whose DNA sample passed genetic quality control comprised the analytical sample. Anthropometric and BP measurements were obtained at cycles 1, 12, 19, 22, and 23 when participants were age 13, 15, 17, 24, and 31years on average, respectively. Analyses excluded cycle 23 to focus on participants with an age range closer to that observed in SYS.

Informed consent was obtained from parents at inception and from participants post-high school when they had attained legal age. Separate consent was obtained for collection of blood and saliva samples. This study was approved by the ethics review committees of the Montréal Department of Public Health, the McGill University Faculty of Medicine, and the Centre de Recherche du Centre Hospitalier de l’Université de Montréal.

### Outcome Variables

Trained technicians measured height and weight (Seca Portable Stadiometer – Model 214 and Seca Scale – Model 761, Seca Corporation, Columbia, MD, United States) according to a standardized protocol (Evers and Hooper, 1995). BMI was calculated as weight (kg) divided by height squared (m^2^). BP was assessed three times at 1-min intervals with an oscillometer (Dinamap XL, CR9340, Critikon Co, Tampa, Fla) calibrated to a mercury sphygmomanometer. SBP was computed as the average of the second and third measurements. Additional information on BMI and SBP measurements, such as how measures were taken, was reported previously (O’Loughlin et al., 2014) and can also be found in Supplementary Text 1.

### SNP Genotyping

In addition to the three SNPs associated with both adiposity and SBP in SYS [i.e., rs16933812 (*PAX5*), rs7638110 (*MRPS22*), and rs9930333 (*FTO*)], we also investigated two additional SNPs that were associated with adiposity but not SBP in SYS [i.e., rs7120548 (*MTCH2*), rs17773430 (*MC4R*)]. This was performed to further validate that associations with both phenotypes would not be specific to the NDIT sample. Genotyping was performed by Genome Québec using the Illumina Global Screening Array-24 v1.0 (GSA) and imputation was done with Minimac3 software (Das et al., 2016) according to haplotypes from the 1,000 Genomes Phase 3 reference panel (Auton et al., 2015). The SNP rs17773430 (*MC4R*) was not available within the genotyping array used and was therefore replaced with a proxy, rs111638368 (*MC4R*), in strong linkage disequilibrium (LD; r^2^=0.976) in a comparable population to NDIT [i.e., Utah residents with Northern and Western European ancestry (CEU)] in the 1,000 Genomes Project Phase 3 (Machiela and Chanock, 2015). Additional details on the genotyping process and genetic quality control are available in Supplementary Text 2.

In SYS, SNPs rs9930333 (*FTO*), rs7120548 (*MTCH2*), and rs111638368 (*MC4R*) were selected to be tested for their association with SBP based on their strong association with BMI (*p* < 5×10^−4^) and because they were in high LD (D′ > 0.90) with at least one of 33 SNPs previously identified in published GWAS for BMI [respectively rs9939609 (*FTO*), rs3817334 (*MTCH2*), rs17782313 (*MC4R*)]. LD measures were calculated in NDIT to examine potential discrepancies with the values observed in SYS (Table 1). Consistent with SYS, high LD was observed in NDIT for the *FTO* (D′=0.96 for rs9930333 and rs9939609) and *MTCH2* SNPs (D′=1.00 for rs7120548 and rs3817334). In contrast, rs111638368 (*MC4R*) was in low LD with rs17782313 (*MC4R*) in NDIT (D′=0.33). To assess whether the difference in LD between SYS and NDIT could lead to differences in the strength of associations with BMI and SBP, we included both rs111638368 (*MC4R*) and rs17782313 (*MC4R*) in the main analysis. Additionally, LD patterns at both rs16933812 (*PAX5*) and rs7638110 (*MRPS22*) locus observed in NDIT were comparable to those observed in SYS (Supplementary Figures 1, 2).

**Table 1:** Measure of linkage disequilibrium (LD; D') between single nucleotide polymorphisms (SNPs) previously reported in genome-wide association study (GWAS), Melka et al. (2012) and Nicotine Dependence In Teens (NDIT) studies.

GWAS			SYS		NDIT		
Gene	SNP^1^	References	SNP^1^	D'	SNP^1^	D'	R^2^
FTO	rs9939609	Willer et al., 2009	rs9930333	>0.90	rs9930333	0.96	0.82
MTCH2	rs3817334	Speliotes et al., 2010	rs7120548	>0.90	rs7120548	1.00	0.31
MC4R	rs17782313	Loos et al., 2008	rs17773430	> 0.90	rs111638368	0.33	0.08

1 SNP investigated in the original GWAS study, in SYS and in NDIT.

### Data Analysis

Characteristics of the 713 participants retained in the NDIT sample were described at each cycle. Normally distributed variables were expressed as mean (SD), non-normally distributed variables as medians (IQR), and categorical variables as frequencies.

Associations of each SNP investigated with BMI and SBP were estimated using linear mixed models to account for repeated measures. Models were adjusted for age at baseline, sex, time elapsed since baseline (0, 36, 57, and 144months), and height (SBP only) and a compound symmetry correlation structure was assumed for the error terms. The fit of the models under alternative correlation structures were compared in sensitivity analyses, but the results did not warrant we re-examine our initial selection (Supplementary Table 2; Supplementary Text 3). Genotype was coded according to a genotypic model (i.e., minor allele homozygous, heterozygous, or major allele homozygous) to compare CIs to those estimated in SYS. Because an additive model has more power to detect genetic associations, additive coding was assessed in sensitivity analyses for all SNPs for both BMI and SBP. The analysis of residuals (Supplementary Figures 3–8) did not suggest any important violations of the assumptions of normality, linearity, or homogeneity of variance required for valid inference in the linear mixed models (Jacqmin-Gadda et al., 2007). All statistical analysis were conducted with R statistical software version 3.6.3 (R Core Team, 2019) and all LD calculations and mapping in NDIT were conducted using Haploview v4.2 (Barrett et al., 2004).

## Results

Table 2 describes participant characteristics in cycles 1, 12, 19, and 22. There were more females than males across all cycles (54–56%). Mean BMI increased over time from 20.2kg/m^2^ at cycle 1 to 24.6kg/m^2^ at cycle 22. Mean BP increased from cycle 1 to 12 but plateaued from cycle 19 to 22. The sample size decreased due to attrition, from 713 in cycle 1 to 601 (84% of 713) in cycle 22. The number of missing values in cycles 1, 12, 19, and 22 are shown in Supplementary Table 3. Compared to participants lost-to-follow-up before cycle 22, those retained were younger, relatively more were female (56 vs. 42%), fewer had ever smoked (29 vs. 39%) and they had a lower BMI (1.2kg/m^2^) on average at baseline (Supplementary Table 4).

**Table 2:** Characteristics of analytical sample at cycles 1, 12, 19, and 22, NDIT 1999–2012^**^.

Variable	Cycle 1 (*n*=713)	Cycle 12 (*n*=712)	Cycle 19 (*n*=688)	Cycle 22 (*n*=601)
Age, mean (SD)	12.7 (0.5)	15.2 (0.5)	17.0 (0.4)	24.0 (0.7)
Female, %	53.7	53.7	54.4	55.9
Mother university-educated, %	45.5	45.5	45.9	46.3
Canada born, %	98.5	98.5	98.7	98.8
Single-parent family, %	6.3	11.6	14.8	
Alcohol consumption, %	45.4	54.9	77.2	
Depression symptoms, mean (SD)	2.1 (0.6)	2.0 (0.8)	2.1 (0.8)	
Ever smoked, %	30.2	57.7	64.1	81.0
No cigarettes/month, med (IQR)	0.2 (1.7)	0.0 (6.6)	0.0 (8.3)	0.2 (112.5)
Height, mean (SD)	156.3 (7.7)	166.9 (7.9)	169.6 (8.6)	170.4 (9.0)
BMI, mean (SD)	20.2 (3.8)	21.9 (3.7)	22.7 (3.7)	24.6 (4.6)
SBP, mean (SD)	105.4 (10.1)	106.8 (10.4)	110.8 (11.3)	109.2 (11.5)

n, sample size; SD, standard deviation; IQR, interquartile range; and med, median.

** For each cycle *n*=number of participants remaining in the study. At cycle 22, data for family composition, depression symptoms, and alcohol consumption were not collected, while body mass index (BMI) z-score was not considered since participants were adults. No cigarettes/month: number of cigarettes smoked per month for the last 3months among ever smokers. Additional details on variables and how they were coded is available in Supplementary Table 1.

Table 3 compares SYS results to those obtained in NDIT for BMI and SBP. Minor allele frequencies (MAFs) were similar across studies except for rs16933812 (*PAX5*), which was higher in NDIT than in SYS (37 vs. 22%). Of the three SNPs associated with both adiposity and SBP in SYS, only rs9930333 (*FTO*) was significantly associated with BMI in NDIT. The mean BMI difference between minor allele homozygotes and major allele homozygotes for rs9930333 (*FTO*) was 1.18kg/m^2^ (95% CI 0.42; 1.94). The mean difference in SBP between minor allele homozygotes and major allele homozygotes for this SNP was not significant [i.e., 0.64mmHg (95% CI −1.08; 2.35)]. Neither rs16933812 (*PAX5*) nor rs7638110 (*MRPS22*) were associated with BMI or SBP in NDIT. The estimated difference in BMI for the *PAX5* SNP was close to zero with a narrow CI and was in the opposite direction compared to SYS for the *MRPS22* SNP. The CIs for the estimated association between both the *PAX5* and *MRPS22* SNPs with SBP included 0 and both were in the opposite direction in NDIT compared to SYS.

**Table 3:** Estimated coefficients of the investigated SNPs in their association with BMI and SBP, NDIT 1999–2012 and Melka et al. (2012) 2003–2009^**^.

SNP information			SYS			NDIT				
Gene	SNP	Minor allele	MAF	B̂ [CI]	*p* value	Proxy	Minor allele	MAF	B̂ [CI]	*p* value
BMI (*n*=713/2350)										
PAX5	rs16933812	G	0.22	3.3 [1.9, 4.6]	**5.2×10** ^**−6**^	_	G	0.37	−0.03 [−0.88, 0.81]	0.9353
MRPS22	rs7638110	T	0.06	2.8 [1.9, 3.6]	**4.6×10** ^**−8**^	_	T	0.07	−0.22 [−0.98, 0.53]	0.5623
FTO	rs9930333	G	0.38	1.8 [NA]	**1.9×10** ^**−4**^	_	G	0.43	1.18 [0.42, 1.94]	**0.0025**
MTCH2	rs7120548	C	0.29	−1.6 [NA]	**1.9×10** ^**−4**^	_	C	0.35	−0.89 [−1.76, −0.02]	**0.0445**
MC4R	rs17773430	C	0.27	2.4 [NA]	**5.8×10** ^**−6**^	rs111638368	T	0.29	0.71 [−0.27, 1.68]	0.1565
MC4R	rs17782313	_	_	_	_	_	C	0.23	1.18 [0.04, 2.32]	**0.0431**
SBP (*n*=713/2347)										
PAX5	rs16933812	G	0.22	6.68 [2.80, 10.57]	**0.0007**	_	G	0.37	−0.55 [−2.44, 1.34]	0.5716
MRPS22	rs7638110	T	0.06	3.30 [0.49, 6.12]	**0.02**	_	T	0.07	−1.52 [−3.20, 0.17]	0.0777
FTO	rs9930333	G	0.38	3.63 [0.73, 6.53]	**0.01**	_	G	0.43	0.64 [−1.08, 2.35]	0.4679
MTCH2	rs7120548	C	0.29	−0.51 [−3.86, 2.84]	0.77	_	C	0.35	0.31 [−1.65, 2.26]	0.7563
MC4R	rs17773430	C	0.27	−0.05 [−3.44, 3.34]	0.98	rs111638368	T	0.29	0.77 [−1.41, 2.95]	0.4880
MC4R	rs17782313	_	_	_	_	_	C	0.23	1.96 [−0.60, 4.53]	0.1337

*n*, number of participants/total observations; MAF, minor allele frequency; and SYS, Saguenay Youth Study (Pausova et al., 2017).

** Estimated differences in SBP and BMI levels of minor allele vs. major allele homozygous (*PAX5*, *MTCH2*, *MC4R*, and *FTO*) and minor allele homozygous+heterozygous vs. major allele homozygous (*MRPS22*) with 95% CI. BMI 95% CI not reported for SNPs at *FTO*, *MTCH2*, and *MC4R* genes in Melka et al. (2012) BMI *values of p* reported for SYS corresponds to the linear regression GWAS, where genotype is coded additively. Sample size corresponds to *n*=number of participants/number of observations with available trait (BMI or SBP) data. *Values of p* were obtained from Student’s *t*-test. Bold *p*-value signifies a statistically significant (*p* < 0.05) difference in BMI or SBP.

Consistent with SYS, SNP rs7120548 (*MTCH2*) was associated with BMI, but not SBP in NDIT. The mean BMI difference between minor allele homozygotes and major allele homozygotes was −0.89kg/m^2^ (95% CI −1.76; −0.02) for rs7120548 (*MTCH2*). The *MC4R* SNP rs111638368 investigated in SYS was not significantly associated with BMI, while the previously identified *MC4R* SNP rs17782313 showed a greater difference in BMI than rs111638368 and was statistically significant. The mean BMI difference between minor allele homozygotes and major allele homozygotes was 0.71kg/m^2^ (95% CI −0.27; 1.68) for rs111638368 (*MC4R*) and 1.18kg/m^2^ (95% CI 0.04; 2.32) for rs17782313 (*MC4R*).

Additional sensitivity analyses were done to verify robustness to the various model specifications used and results are available in Supplementary Text 3, Supplementary Tables 5–9, and Supplementary Figure 9. Overall, these analyses revealed that our results are robust to the genotype coding model, the use of BMI compared to BMI z-score as the adiposity measure, the use of young adults in our analytical sample, and to potential confounding by population stratification. Because BMI is a strong predictor of SBP in youth (Chorin et al., 2015), we re-estimated the SBP models, this time adjusting for BMI. Among the three SNPs associated with SBP in SYS (i.e., *FTO*, *PAX5*, and *MRPS22*), adjusting for BMI did not change the estimated coefficient of the *PAX5* SNP, while the estimated coefficients for *FTO* and *MRPS22* decreased toward the null, suggesting that BMI might be an intermediary variable between these two SNPs and SBP.

## Discussion

Investigating the genetic underpinnings of adiposity and BP in youth is important because it cannot be assumed that the genetic architecture in adults is applicable in children and adolescents (Wang et al., 2015). Further, investigating genetic susceptibility in youth, who are rarely treated with antihypertensive medication and who are less affected than adults by the cumulative impact of exposure to the environment, might help identify genetic factors that influence both adiposity and BP (Bradfield et al., 2012; Parmar Priyakumari et al., 2016). Consistent with SYS, we replicated the association of rs9930333 on *FTO* with BMI. This observation aligns with several other studies in children and adolescents that observed an association between SNPs on *FTO* and adiposity traits, such as BMI (den Hoed et al., 2010; Felix et al., 2016), BMI z-score (Namjou et al., 2013), and obesity (Zhao et al., 2011; Bradfield et al., 2012; da Silva et al., 2018). We did not replicate the associations of SNPs rs16933812 and rs7638110 on *PAX5* and *MRPS22* with BMI. Additionally, none of the three associations with SBP were replicated. Overall, our results do not support the hypothesis that the variants investigated in Melka et al. (2012) contribute to the correlation between BMI and SBP levels in youth. Potential explanations for the lack of replication are reviewed, including statistical considerations and genetic and phenotypic differences across studies.

### Statistical Considerations

First, several genetic associations reported in Melka et al. (2012), could represent spurious findings that cannot be replicated in independent datasets. Two SNPs associated with BMI and SBP in Melka et al. (2012) [i.e., rs16933812 (*PAX5*), rs7638110 (*MRPS22*)] were novel discoveries that were not associated with either adiposity or BP in the most recent GWAS for each trait (Evangelou et al., 2018; Yengo et al., 2018). Melka et al. (2012) used the same dataset for discovery and effect estimation, which may lead to overestimation of genetic effects (Göring et al., 2001). Further, Melka et al. (2012) used a two-stage procedure in which several SNPs were first selected conditional on the *value of p* of their association with BMI, before their association with SBP was assessed. This could also lead to an overestimation of associations because estimated effects are strongly correlated with *p*-values (Faye et al., 2011). Supporting this, coefficients estimated in SYS for the association of novel SNPs rs16933812 (*PAX5*) and rs7638110 (*MRPS22*) on BMI were higher compared to those for known BMI SNPs rs9930333 (*FTO*), rs7120548 (*MTCH2*), or rs17773430 (*MC4R*) and thus, would be expected to have been discovered in previous studies with much larger sample sizes.

Although, some GWAS have detected associations between rs9930333 (*FTO*) and BP traits (Feitosa et al., 2018; Sung et al., 2018; Takeuchi et al., 2018), the most recent consortiums have not identified such an association (Hoffmann et al., 2017; Evangelou et al., 2018). Inconsistencies between SYS and NDIT for the estimated associations between SNP rs9930333 (*FTO*) and SBP may be due to the potential role of BMI as an intermediate variable. BMI is a known predictor of SBP in adolescents (Chorin et al., 2015) and thus, in the absence of adjustment for BMI, the association between *FTO* and SBP could solely capture the indirect effect of *FTO* on SBP through BMI. In NDIT, the coefficient of association between rs9930333 (*FTO*) and SBP, although not significant, was reduced to zero after adjusting for BMI (Supplementary Table 6), suggesting that BMI could be an intermediate variable responsible for the association between *FTO* and SBP in SYS. In another study using SYS participants (Pausova et al., 2009), adjusting for BMI reduced the effect of rs9939609 (*FTO*) on SBP, although it remained significant. The association between these SNPs on *FTO* and SBP warrants additional investigation, especially considering the potential role of BMI in the association.

### Genetic Differences Across Studies

Second, differences in LD pattern between populations can result in inconsistencies in genetic effects, since most SNPs detected in genetic association studies are not causal for the trait investigated but are in high LD with a causal SNP. Although both samples were of European ancestry, SYS participants comprised adolescents from a founder population in Saguenay-Lac-St-Jean (Pausova et al., 2017), in contrast to the admixed NDIT sample in Montréal, Canada. Founder populations usually show a higher degree of LD than admixed populations (Service et al., 2006). In SYS, rs17773430 (*MC4R*) was in high LD (D′ ˃ 0.9) with rs17782313 (*MC4R*), a SNP previously identified for its association with BMI in a GWAS (Loos et al., 2008). However, LD measures between these two SNPs are much lower in the 1,000 Genomes Project Phase 3 [D′=0.31 using the Utah residents with Northern and Western European ancestry (CEU); Machiela and Chanock, 2015], which closely resembled the LD between rs17782313 (*MC4R*) and a proxy of rs17773430 (*MC4R*) in NDIT. This suggests that the difference in LD observed between SYS and NDIT likely reflects a population-specific LD pattern in the Saguenay-Lac-St-Jean founder population. This difference in LD likely explains the non-replication of the association between the proxy of rs17773430 (*MC4R*) and BMI in NDIT, as rs17782313 (*MC4R*) was associated with BMI in NDIT.

Studies have suggested that physical activity (Kilpeläinen et al., 2011; Young et al., 2016; Graff et al., 2017), alcohol consumption (Young et al., 2016), diet (Young et al., 2016), and sleep duration (Young et al., 2016) modify the associations between variants on *FTO* and BMI. Also, stronger associations between variants on *FTO* and SBP has been observed in alcohol users compared to non-users (Feitosa et al., 2018). The strength of the associations estimated in different samples can vary because of gene–environment interactions when the environmental factors vary in their distributions between samples (Schielzeth et al., 2018). Although such differences between SYS and NDIT could not be assessed, the age range in the two samples was similar and further, participants lived in the same geographic location, which limits the likelihood that gene–environment interactions explain the lack of replication.

### Differences in Phenotypes Across Studies

Third, differences in BP measurement techniques may contribute to failed replication of the associations between rs16933812 (*PAX5*), rs7638110 (*MRPS22*), and rs9930333 (*FTO*) and SBP. Average BP in SYS was measured in a 52-min protocol including measures at different postures (supine, standing, and sitting) and different levels of stress (during mental stress and during mental stress recovery; Pausova et al., 2017). In NDIT, BP was measured in sitting position only according to a standard protocol (O'Loughlin et al., 2015). Short-term variation in BP in response to position change or mental effort is regulated by multiple factors including sympathetic outflow, cardiopulmonary reflexes, blood vessel elasticity, blood viscosity and hormones, and the genetic architecture of this variation remains largely unknown (Parati et al., 2013). Inconsistencies in associations with SBP across studies may reflect heterogeneity of BP sub-phenotypes related to differences in measurement.

Limitations of this study include lack of power to detect the effect of low MAF SNPs and the potential for selection bias. Specifically, NDIT had less power to detect SNP rs7638110 (*MRPS22*), which has the MAF of only 7% compared to the other SNPs investigated, which have MAFs over 20%. Although retention in NDIT was good (i.e., 84% of participants at inception were available in cycle 22), loss-to-follow-up could still induce selection bias. Although debated, selection bias can occur in longitudinal genetic association studies when the genetic variants studied are associated with a secondary phenotype which affects retention in the study (Munafò et al., 2018).

In conclusion, we replicated two associations observed in an earlier study of Quebec adolescents of European ancestry between SNPs in the well-established adiposity genes *FTO* and *MTCH2* and BMI. However, we could not replicate SNPs at genes *PAX5*, *MRPS22*, or *MC4R*. None of the SNPs showed an association with SBP. Our findings underscore again that initial discovery of genetic associations should be interpreted cautiously. Since lack of genetic data on youth limits the possibility of large sample size genetic association studies, replication of smaller analyses in comparable study population remains crucial to inform the genetic architecture of youth adiposity and BP.

## Data Availability Statement

The data analyzed in this study is subject to the following licenses/restrictions: NDIT data. The datasets generated and/or analyzed during the current study are not publicly available due to participant confidentiality issues but are available from the Principal Investigator (jennifer.oloughlin@umontreal.ca) upon successful completion of the application process. Requests to access these datasets should be directed to jennifer.oloughlin@umontreal.ca.

## Ethics Statement

The studies involving human participants were reviewed and approved by Ethics review committees of the Montréal Department of Public Health, the McGill University Faculty of Medicine, and the Centre de Recherche du Centre Hospitalier de l’Université de Montréal. Informed consent was provided at inception by participants’ legal guardians. Informed consent was provided by participants (who had attained legal age) post high school.

## Author Contributions

DG wrote the initial draft, and was responsible for conceptualization, formal analysis, and methodology. JO’L did review and editing. M-PS did review and editing and was responsible for conceptualization, methodology, and supervision. All authors contributed to the article and approved the submitted version.

## Conflict of Interest

The authors declare that the research was conducted in the absence of any commercial or financial relationships that could be construed as a potential conflict of interest.

## Publisher’s Note

All claims expressed in this article are solely those of the authors and do not necessarily represent those of their affiliated organizations, or those of the publisher, the editors and the reviewers. Any product that may be evaluated in this article, or claim that may be made by its manufacturer, is not guaranteed or endorsed by the publisher.

## Abbreviations

BP: Blood pressure
BMI: Body mass index
SBP: Systolic blood pressure
DBP: Diastolic blood pressure
SNP: Single nucleotide polymorphism
NDIT study: Nicotine dependence in teens study
SYS: Saguenay Youth Study
LD: Linkage disequilibrium
MAF: Minor allele frequency
