# Supplementary material for: Association of Genetic Variants With Body-Mass Index and Blood Pressure in Adolescents: A Replication Study
Source: Front Genet. 2021 Sep 1;12:690335. doi: 10.3389/fgene.2021.690335 (PMC8440872; doi:10.3389/fgene.2021.690335)
Supplement: Supplementary file 1 [file Data_Sheet_1.docx]

**SUPPLEMENTARY MATERIAL**

**Supplementary Table 1: Detailed description of questionnaire study variables**

| **Variable** | **Variation over time** | **Used cycle(s)** | **Available in cycle(s)** | **Item(s)** | **Response choice** | **Recoding for analysis** |
| --- | --- | --- | --- | --- | --- | --- |
| Age | Yes | 1, 12, 19, 22 | 1-23 | Date of birth, date of survey (1-20), today's date (21-23) | As is | - |
| Sex | No | 1 | 1-11 | Are you a boy or a girl? | Male, female |  |
| Mother university-educated | No | 9 (school 10 only), 13, 17 and mother questionnaire | 9 (school 10 only), 13, 17 and mother questionnaire | How much education has your mother had? | did not finish high school, high school graduate, vocational, technical school, CEGEP, university, don't know, not applicable, other | No, yes (mother attended university, even if not graduated) |
| Country of birth | No | 1 | 1-11 | Were you born…? | in Canada (name province), outside Canada (name country) | - |
| Single family | Yes | 1, 12, 19 | 1-20 | This chart asks about the adults with whom you live. Check the box if you live with the person…If you live in more than one household (part time with your Mom and part time with your Dad), check ALL the boxes that apply (i) biologic mother, (ii) biologic father, (iii) stepmother, (iv) step-father, (v) aunt(s), (vi) uncle(s), (vii) grandmother(s), (viii) grandfather(s), (ix) other(s) (name them) | No, yes (for each person) | No, yes (only one adult) |
| Alcohol consumption in the past 3 months | Yes | 1, 12, 19 | 1-20 | During the past 3 months, how often did you…? (i) smoke a cigar or cigarillo, (ii) use chewing tobacco or snuff, **(iii) drink alcohol (beer, wine, hard liquor)** | never, a bit to try, once or a couple of times a month, once or a couple of times a week, every day | No, yes (a bit to try to more) |
| Depression symptoms | Yes | 1, 12, 19 | 1-20 | During the past 3 months how often have you:  felt too tired to do things,  had trouble going to sleep or staying asleep,  felt unhappy, sad or depressed,  felt hopeless about the future,  felt nervous or tense,  worried too much about things | Never, Rarely,  Sometimes,  Often | Depression symptoms = Sum of 6 items (1 to 4) divided by number of items responded to, to create a continuous score (range 1 to 4) |
| Ever smoked cigarette | Yes | 1, 12,19, 22 | 1-23 | Indicators of frequency and intensity of smoking:  Cycles 1-20: Have you ever in your life smoked a cigarette, even just a puff (drag, hit, haul)?  And  Check the one box that describes you best…  Cycle 22: Have you ever in your life smoked a cigarette, even just a puff (drag, hit, haul)? | Cycle 1-20: No  Yes, 1 or 2 times  Yes, 3 or 4 times  Yes, 5 to 10 times  Yes, more than 10 times  And  I have never smoked  I have smoked but not in the last year  I have smoked once or a couple of times last year  I smoke once or couple of times each month  I smoke once or couple of times each week  I smoke every day  Cycle 22: No  Yes, 1 or 2 times  Yes, 3 or 4 times  Yes, 5 to 10 times  Yes, more than 10 times | No, yes (if one indicator of frequency and intensity of smoking is ≠ 0 during follow-up) |
| Mean number of cigarettes smoked in the past 3 months | Yes | 1, 12, 19, 22 | 1-23 | For each of the past 3 months: During ______, on how many days did you smoke cigarettes, even just a puff?  On the days that you smoked during last month, how many cigarettes did you usually smoke each day? | 0, 1, 2-3, 4-5, 6-10, 11-15, 16-20, 21-30, every day, don’t know  <1, 1, 2-3,4-5, 6-10, 11-15, 16-20, 21-25, >25, don’t know | Number of days was multiplied by number of cigarettes smoked per day and averaged across months |

**Supplementary Text 1: Blood pressure and body-mass index measurement**

Anthropometric and blood pressure measurements were obtained at study cycles 1, 12, 19, 22 NS 23 where participants were respectively 13, 15, 17, 24 AND 31 years old on average. Trained technicians measured height and weight (Seca Portable Stadiometer – Model 214 and Seca Scale – Model 761, Seca Corporation, Columbia, MD, USA) according to a standardized protocol (Evers and Hooper, 1995). Two measures of height and weight were taken at each collection point. BMI was then calculated as weight (kg) divided by height squared (m^2^). A third measure was obtained if discrepancies of 0.2 kg and 0.5 cm was observed and BMI was then computed as the mean of the two closest measures.

Blood pressure was assessed three times with an oscillometer (Dinamap XL, modèle CR9340, Critikon Co, Tampa, Fla) calibrated to a mercury sphygmomanometer at 1-min interval, at the right arm, sitting and after a five-minute rest period. SBP was computed as the average of the second and third SBP measurements. When discrepancies of 20 mmHg SBP or 10 mmHg DBP were observed, fourth and fifth measures were taken and SBP was computed as the average of the two closest measures.

**Supplementary Text 2: Genotyping and genetic quality control procedures**

Genotypes for SNPs of interest were obtained by a genome-wide screening performed on samples from 943 NDIT participants by Genome Québec in 2017 using an Illumina Global Screening Array-24 v1.0 (GSA). Genetic imputations were performed using Minimac3 software (Das et al., 2016) using the 1000 Genome Phase 3 reference panel (Auton et al., 2015). A total of 40 participants had to be removed because of DNA samples of poor quality or sex inconsistencies. From the remaining 903 participants, 868 passed quality control based on a sample call rate threshold of 98%. 726 of those were identified to be of European ancestry based on principal components analysis. Finally, 9 first degree relatives were excluded, bringing the available sample for our study to 717 participants with genotype data.


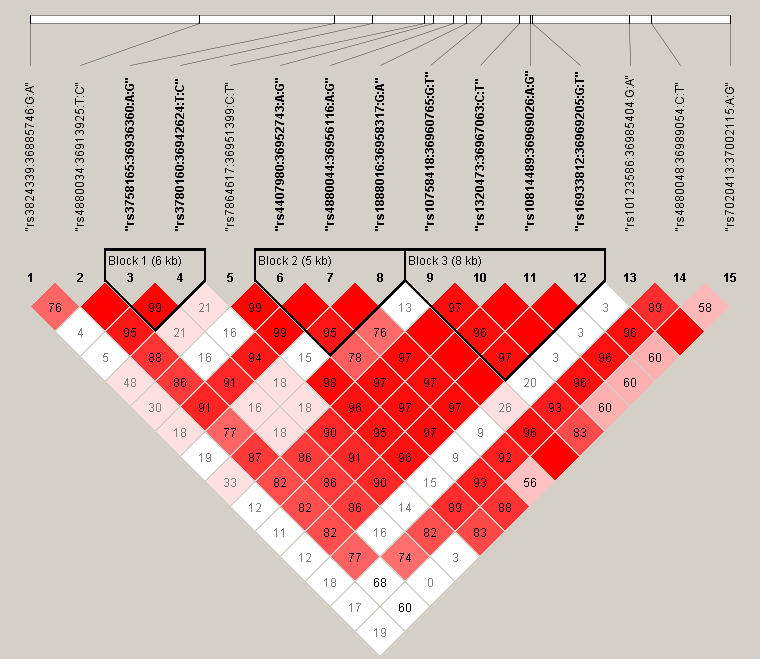


**Supplementary Figure 1. LD (D’) pattern at *PAX5* on chromosome 9**


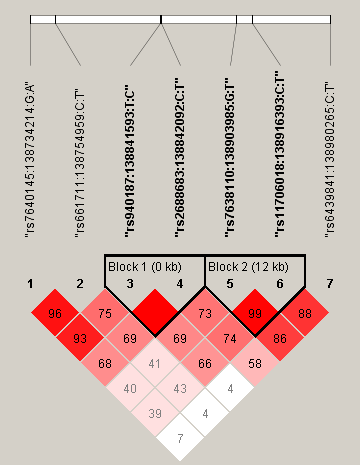


**Supplementary Figure 2. LD (D’) pattern at *MRPS22* on chromosome 3**


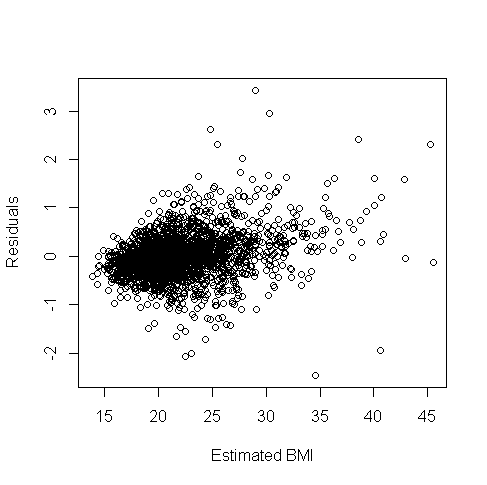


**Supplementary Figure 3. Residuals of final BMI model plotted against estimated BMI**


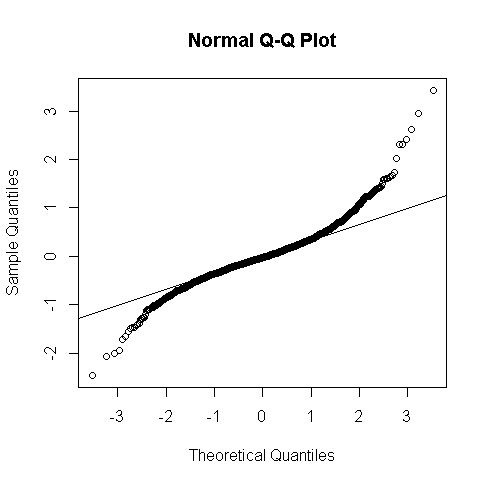


**Supplementary Figure 4. QQplot of final BMI model**


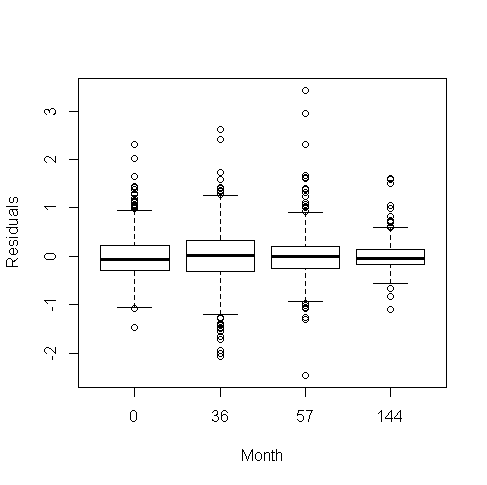


**Supplementary Figure 5. Boxplot of residuals of final BMI model over time**


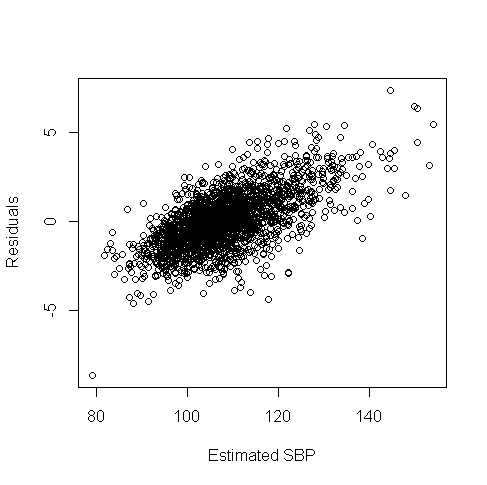


**Supplementary Figure 6.** **Residuals of final SBP model plotted against estimated SBP**

**
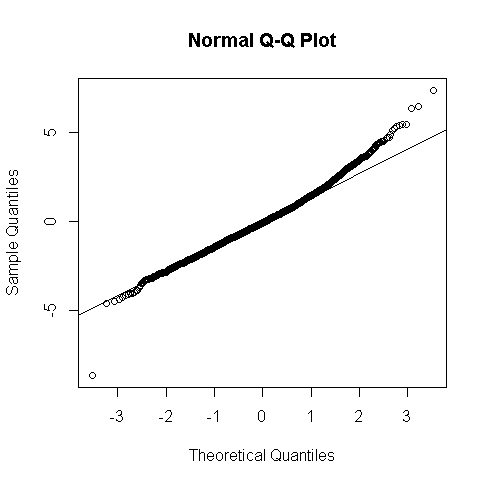
**

**Supplementary Figure 7. QQplot of final SBP model**


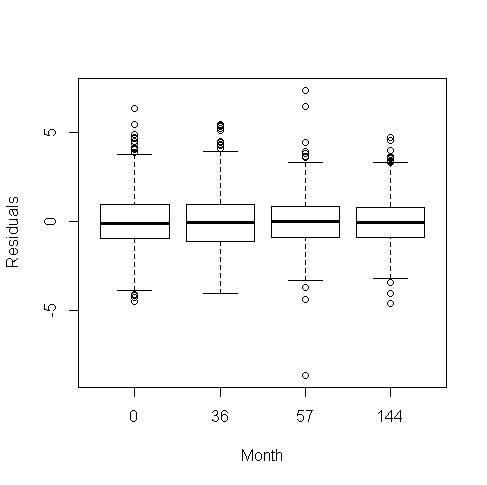


**Supplementary figure 8. Boxplot of residuals of final SBP model over time**

**Supplementary Table 2: Robustness to specification of the correlation structure**

| **Correlation structure** | **AIC**  **(BMI)** | **AIC**  **(SBP)** |
| --- | --- | --- |
| Compound symmetry | 11 070 | 16 950 |
| Autoregressive process of order 1 | 11 052 | 16 947 |

Linear mixed models we specified prior to inclusion of SNPs as fixed effects and are adjusted for age at baseline, sex, time elapsed since baseline and height (SBP only).

**Supplementary Table 3: Missing values at cycles 1, 12, 19 and 22, NDIT 1999-2012**

| **Variable** | **Cycle 1**  **(n = 713)** | **Cycle 12**  **(n = 712)** | **Cycle 19**  **(n = 688)** | **Cycle 22**  **(n = 601)** |
| --- | --- | --- | --- | --- |
| Age, mean (SD) | 0 | 0 | 0 | 0 |
| Female, % | 0 | 0 | 0 | 0 |
| Mother university-educated, % | 73 | 72 | 60 | 46 |
| Canada born, % | 0 | 0 | 0 | 0 |
| Single-parent family, % | 32 | 93 | 142 | - |
| Alcohol consumption, % | 37 | 102 | 145 | - |
| Depression symptoms, mean (SD) | 35 | 101 | 142 |  |
| Ever smoked, % | 14 | 88 | 136 | 0 |
| No cigarettes/month, med (IQR) | 8 | 10 | 11 | 7 |
| Height, mean (SD) | 48 | 106 | 166 | 44 |
| BMI, mean (SD) | 48 | 106 | 166 | 44 |
| SBP, mean (SD) | 49 | 104 | 167 | 45 |

Note: n = sample size, SD = standard deviation, IQR = interquartile range, med = median.

No cigarettes/month: number of cigarettes smoked per month for the last three months among ever smokers. Data were missing because participants not completing a questionnaire or missing data on specific variables.

**Supplementary Table 4: Comparison of baseline characteristics of participants remaining and lost to follow-up, NDIT 1999**

| **Variable** | **Remaining**  **(n=601)** | **Attrited**  **(n=112)** | **p value** |
| --- | --- | --- | --- |
| Age, mean (SD) | **12.7 (0.4)** | **12.8 (0.6)** | **0.001** |
| Female, % | **55.9** | **42.0** | **0.009** |
| Mother university-educated^1^, % | 46.3 | 40.0 | 0.332 |
| Single family, % | 6.2 | 6.8 | 1.000 |
| Alcohol consumption^2^, % | 44.1 | 52.9 | 0.121 |
| Depression symptoms, mean (SD) | 2.1 (0.6) | 2.1 (0.7) | 0.877 |
| Ever smoked, % | **28.5** | **39.4** | **0.029** |
| No cigarettes/month, med (IQR) | 0.2 (2.1) | 0.0 (0.4) | 0.357 |
| Height, mean (SD) | 156.1 (7.5) | 156.8 (8.8) | 0.407 |
| BMI, mean (SD) | **20.0 (3.8)** | **21.2 (4.0)** | **0.003** |
| SBP, mean (SD) | 105.2 (9.8) | 106.2 (11.6) | 0.373 |

Note: n = sample size, SD = standard deviation, IQR = interquartile range, med = median.

No cigarettes/month: number of cigarettes smoked per month for the last three months among ever smokers. For categorical variables differences between included and excluded participants were assessed using chi-square. Differences in means of normally distributed continuous variables were assessed with ANOVA. Differences in means of non-normally distributed continuous variables (no cigarettes/month) were assessed with Wilcox-test

**Supplementary Text 3: Summary of sensitivity analyses**

1) **Specification of the correlation structure**: We compared the fit of the model that used a compound symmetry correlation structure with that of a model that used an autoregressive process of order 1 in Supplementary Table 2. Even though an autoregressive process of order 1 structure had a slightly better fit than a compound symmetry correlation structure for both SBP and BMI models, we chose to retain the latter to account for the longitudinal aspect of our repeated outcome measures, separated by a non-equivalent time interval (0, 36, 57, 144 months since baseline).

2) **Additive coding model of alleles**: As shown in Supplementary Table 5, results from the additive models (0, 1 or 2 alleles) were aligned with those obtained under a genotypic model. SNPs at the *FTO* and *MTCH2* genes were the only two variants associated with BMI. Each additional minor allele at rs9930333 (*FTO*) was associated with a 0.61 (95% CI 0.24 ; 0.99) kg/m^2^ increase in BMI. Each additional minor allele at rs7120548 (*MTCH2*) was associated with a 0.44 (95% CI 0.05-0.84) kg/m2 decrease in BMI. Because an additive model provided a better fit based on AIC and because statistically significant associations identified were graphically linear, additive coding was preferred for following sensibility analyses.

3) **Adjusting for BMI in SBP models**: Because the SNPs investigated for their association with SBP were selected based on their association with BMI and that BMI is a predictor of SBP, we considered adjusting for BMI and BMI^2^ in the SBP models. Squared BMI was never associated significantly with SBP. In the NDIT sample, BMI is found to be strongly associated with SBP (5.47 X 10^-28^ ≤ p ≥ 1.38 X 10^-27^). Adding BMI to SBP models seems to either have little to no effect on the association between SNPs investigated (*PAX5* and *MRPS22* SNPs) and SBP or to mediate, at least in part, the association of the SNPs with SBP (*FTO*, *MTCH2* and *MC4R* SNPs) (Supplementary Table 6). However, as in the models without adjustment for BMI, none of the SNPs investigated are significantly associated with SBP in the models adjusting for BMI. Adjusting for BMI but not height resulted in BMI having a slightly stronger association with SBP than observed in our model that adjusted for both BMI and height (β_BMI_ = 0.77 mm Hg vs 0.70 mm Hg). Further, both models yielded similar associations between each SNP and SBP (i.e., our interpretation of the results did not change).

4) **Adjusting for SNP X Time interaction in BMI and SBP models**: To investigate if time could modify the association of SNPs with BMI and SBP, an interaction term between SNPs and time elapsed since baseline was considered. We identified a statistically significant time by SNP interaction for rs111638368 (*MC4R*) (Supplementary Table 7). However, the SNP’s additive effects at each cycle time points all included 0, meaning rs111638368 (*MC4R*) does not substantially affect SBP from adolescence to young adulthood.

5) **Adjusting for SNP X BMI interaction in SBP models**: Because previous studies (Xi et al., 2013; Kim et al., 2014) suggested that adiposity could modify the association between SNPs and blood pressure, an interaction term between SNPs and BMI was considered to examine if BMI could modify the effect of SNPs on SBP. The estimated coefficient of the interaction term between rs111638368 (*MC4R*) and BMI was significantly associated (p = 0.0410) with SBP. Supplemental Figure 9 shows the estimated additive effect of rs111638368 on SBP conditional on BMI. While the estimated effect of rs111638368 on SBP increases with BMI level, its 95% CI (in grey) includes 0 for all BMI values below 35, which corresponds to only 25 individuals (3,5%) in our sample who had at least one value of BMI over 35 kg/m^2^ over the course of the study. Incidentally, rs111638368 only showed an effect on SBP only for a narrow range of BMI values referring to extrapolation of observations gathered in individuals with BMI levels closer to the mean.

Since BMI increases with age over adolescence, the interaction between SNPs and BMI could simply reflect that the estimated effect of the same SNP was seemingly also modified by time. Since the marginal SNP effects on SBP does not evolve similarly when conditioning on BMI compared to Time since baseline, the estimated effect of the SNP on SBP is seemingly independently modified by time and BMI. All interaction analysis were done using the interplot package (Solt and Hu, 2019) in the R statistical software.

6) **Use of BMI z-score to measure adiposity in adolescents**: Using BMI z-score instead of BMI as an outcome of interest did not substantively change the results, suggesting the robustness to the use of BMI as adiposity measurement in adolescence (Supplementary Table 8).

7) **Restriction of analysis to adolescents only**: In order to examine if the inclusion of young adults in our data compared to Melka et al. could affect replication, we restricted the analysis on BMI and SBP to cycles 1, 12 and 19. As shown in Supplementary Table 8, associations were similar across all SNPs and traits under investigation, suggesting that the analysis is robust to the inclusion of young adults.

8) **Principal Component Analysis**: In order to evaluate potential confounding by the presence of sub-ancestry within the analytical sample of European ancestry, we adjusted BMI and SBP linear models with the first five principal components. As shown in Supplementary Table 9, estimated effects adjusted and non-adjusted for the first five principal components are similar. The SNP rs7638110 (*MRPS22*) was statistically significative when adjusting for the components. However, the direction of effect is opposite to the one observed in Melka et al.

**Supplementary Table 5: Beta coefficients and 95% confidence intervals estimated in linear mixed models for the associations between five SNPs and each of BMI and SBP under an additive genetic model, NDIT 1999-2012**

| **Description of SNP** | | | | **BMI (n=713/2350)** | **SBP (n=713/2347)** |
| --- | --- | --- | --- | --- | --- |
| **Gene** | **rsID** | **Minor allele** | **MAF** | **B̂ [CI]** | **B̂ [CI]** |
| PAX5 | rs16933812 | G | 0.37 | -0.01  [-0.40, 0.38] | -0.29  [-1.17, 0.58] |
| MRPS22 | rs7638110 | T | 0.07 | -0.28  [-0.99, 0.43] | -1.52  [-3.10, 0.07] |
| FTO | rs9930333 | G | 0.43 | **0.61**  **[0.24, 0.99]** | 0.41  [-0.43, 1.26] |
| MTCH2 | rs7120548 | C | 0.35 | **-0.44**  **[-0.84, -0.05]** | 0.12  [-0.77, 1.00] |
| MC4R | rs111638368 | T | 0.29 | 0.36  [-0.05, 0.77] | 0.12  [-0.80, 1.04] |
| MC4R | rs17782313 | C | 0.23 | 0.43  [-0.01, 0.86] | -0.08  [-1.06, 0.90] |

Notes: n = analytical sample/number of observations, rsID = identifier

Association analyses was performed with linear mixed models with age at baseline, data collection cycle, sex and height (SBP only) as covariates. Estimated effects correspond to the additive effect of one minor allele increase on BMI and SBP levels with 95% CI. **Bold** effects signify a 95% CI excluding 0.

**Supplementary Table 6: Estimated effects of the five SNPs investigated and BMI on SBP, NDIT 1999-2012**

| **Model** | **Risk factor** | **B̂ [IC]** | **P value** |
| --- | --- | --- | --- |
| **FTO** | | | |
| Main model adjusted for height | rs9930333 | 0.41 [-0.43, 1.26] | 0.34 |
| Adjustment for height and BMI | rs9930333 | -0.04 [-0.85, 0.78] | 0.93 |
|  | BMI | 0.70 [0.58, 0.83] | 1.38 X 10^-27^ |
| Adjustment for BMI only | rs9930333 | -0.10 [-0.92, 0.72] | 0.81 |
|  | BMI | 0.77 [0.64, 0.89] | 2.08 X 10^-31^ |
| **MRPS22** | | | |
| Main model adjusted for height | rs7638110 | -1.52 [-3.10, 0.07] | 0.06 |
| Adjustment for height and BMI | rs7638110 | -1.30 [-2.83, 0.23] | 0.10 |
|  | BMI | 0.70 [0.58, 0.82] | 1.23 X 10^-27^ |
| Adjustment for BMI only | rs7638110 | -1.26 [-2.80, 0.28] | 0.11 |
|  | BMI | 0.76 [0.64, 0.89] | 2.01 X 10^-31^ |
| **PAX5** | | | |
| Main model adjusted for height | rs16933812 | -0.29 [-1.17, 0.58] | 0.51 |
| Adjustment for height and BMI | rs16933812 | -0.29 [-1.14, 0.55] | 0.49 |
|  | BMI | 0.70 [0.58, 0.83] | 8.42 X 10^-28^ |
| Adjustment for BMI only | rs16933812 | -0.20 [-1.05, 0.89] | 0.64 |
|  | BMI | 0.77 [0.64, 0.89] | 1.40 X 10^-31^ |
| **MTCH2** | | | |
| Main model adjusted for height | rs7120548 | 0.12 [-0.77, 1.01] | 0.79 |
| Adjustment for height and BMI | rs7120548 | 0.43 [-0.42, 1.29] | 0.32 |
|  | BMI | 0.71 [0.58, 0.83] | 5.47 X 10^-28^ |
| Adjustment for BMI only | rs7120548 | 0.29 [-0.57, 1.15] | 0.51 |
|  | BMI | 0.77 [0.64, 0.90] | 1.14 X 10^-31^ |
| **MC4R** | | | |
| Main model adjusted for height | rs111638368 | 0.12 [-0.80, 1.04] | 0.79 |
| Adjustment for height and BMI | rs111638368 | -0.12 [-1.01, 0.77] | 0.49 |
|  | BMI | 0.70 [0.58, 0.83] | 8.38 X 10^-28^ |
| Adjustment for BMI only | rs111638368 | -0.03 [-0.92, 0.86] | 0.94 |
|  | BMI | 0.77 [0.64, 0.89] | 1.58 X 10^-31^ |
| **MC4R** | | | |
| Main model adjusted for height | rs17782313 | -0.08 [-1.06, 0.90] | 0.88 |
| Adjustment for height and BMI | rs17782313 | -0.34 [-1.29, 0.61] | 0.48 |
|  | BMI | 0.71 [0.58, 0.83] | 6.49 X 10^-28^ |
| Adjustment for BMI only | rs17782313 | -0.45 [-1.40, 0.50] | 0.36 |
|  | BMI | 0.77 [0.64, 0.90] | 9.48 X 10^-32^ |

**Supplementary Table 7: Additive estimated effect of MC4R locus on SBP by time of measurement, NDIT 1999-2012**

|  | **Cycle 1** | **Cycle 12** | **Cycle 19** | **Cycle 22** |
| --- | --- | --- | --- | --- |
| **B̂ [IC]** | 0.81  [-0.29, 1.90] | -0.47  [-1.67, 0.73] | -1.00  [-2.35, 0.36] | 0.03  [-1.24, 1.29] |

B̂ [IC]: Estimated additive effect of rs111638368 at the MC4R locus on SBP level at cycles 1, 12,19 and 22.

**Supplementary Table 8: Association analysis of the five SNPs investigated with BMI, BMI z-score and SBP, NDIT 1999-2005**

| **SNP information** | | | **BMI z-score (n=704/1793)** | **BMI (n=704/1793)** | **SBP**  **(n=704/1791)** |
| --- | --- | --- | --- | --- | --- |
| **Gene** | **rsID** | **Minor allele** | **B̂ [IC] ^a^** | **B̂ [IC] ^a^** | **B̂ [IC] ^a^** |
| PAX5 | rs16933812 | G | 0.00  [-0.10,0.10] | -0.03  [-0.43,0.36] | -0.14  [-1.07,0.78] |
| MRPS22 | rs7638110 | T | -0.09  [-0.26,0.08] | -0.29  [-1.00,0.42] | -1.58  [-3.25,0.09] |
| FTO | rs9930333 | G | **0.13**  **[0.04,0.22]** | **0.63**  **[0.25,1.00]** | 0.50  [-0.39,1.39] |
| MTCH2 | rs7120548 | C | -0.10  [-0.19,0.00] | **-0.44**  **[-0.84,-0.04]** | 0.10  [-0.84,1.04] |
| MC4R | rs111638368 | T | 0.03  [-0.07,0.13] | 0.37  [-0.04,0.78] | 0.08  [-0.89,1.06] |
| MC4R | rs17782313 | C | 0.05  [-0.05,0.16] | 0.41  [-0.03,0.85] | -0.25  [-1.29,0.78] |

Notes: n = sample size/number of observations.

^a^Association analysis was performed with linear mixed models with age at baseline (SBP and BMI), sex (SBP and BMI) and height (SBP only) as covariates. Estimated effects correspond to the additive effect of one minor allele increase on BMI and SBP levels with 95% confidence interval. **Bold** effects signify a 95% CI excluding 0.

**Supplementary Table 9: Association analysis of the five SNPs investigated with BMI and SBP, adjusted for first five principal components, NDIT 1999-2012**

| **SNPs information** | | | **BMI**  **(n=713/2350)** | | **SBP**  **(n=713/2347)** | |
| --- | --- | --- | --- | --- | --- | --- |
| **Gene** | **rsID** | **Minor allele** | **PC-adj**  **B̂ [IC] ^a^** | **Non-adj**  **B̂ [IC] ^b^** | **PC-adj**  **B̂ [IC] ^a^** | **Non-adj**  **B̂ [IC] ^b^** |
| PAX5 | rs16933812 | G | 0.01  [-0.39, 0.40] | -0.01  [-0.40, 0.38] | -0.31  [-1.20, 0.57] | -0.29  [-1.17, 0.58] |
| MRPS22 | rs7638110 | T | -0.31  [-1.02, 0.41] | -0.28  [-0.99, 0.43] | **-1.67**  **[-3.26, -0.09]** | -1.52  [-3.10, 0.07] |
| FTO | rs9930333 | G | **0.62**  **[0.25, 0.99]** | **0.61**  **[0.24, 0.99]** | 0.37  [-0.47, 1.21] | 0.41  [-0.43, 1.26] |
| MTCH2 | rs7120548 | C | **-0.46**  **[-0.86, -0.07]** | **-0.44**  **[-0.84, -0.05]** | 0.19  [-0.70, 1.08] | 0.12  [-0.77, 1.00] |
| MC4R | rs111638368 | T | 0.35  [-0.06, 0.76] | 0.36  [-0.05, 0.77] | 0.12  [-0.80, 1.04] | 0.12  [-0.80, 1.04] |
| MC4R | rs17782313 | C | 0.44  [0.00, 0.88] | 0.43  [-0.01, 0.86] | -0.10  [-1.09, 0.90] | -0.08  [-1.06, 0.90] |

Notes: n = sample size/number of observations.

Association analysis was performed with linear mixed models. Models adjusted for age at baseline and sex for BMI, and for age at baseline, sex and height for SBP. **Bold** effects signify a 95% CI excluding 0.

^a^Models Adjusted for first five principal components. Estimated effects correspond to the additive effect of one minor allele increase on BMI and SBP levels with 95% confidence interval.

^b^Models not adjusted for first five principal components. Estimated effects correspond to the additive effect of one minor allele increase on BMI and SBP levels with 95% confidence interval.

**
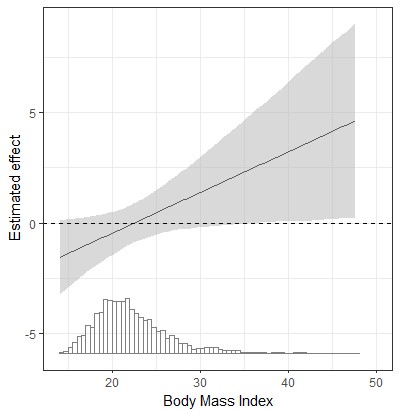
**

**Supplementary Figure 9. Estimated effect of rs111638368 (*MC4R*) on SBP by BMI level, NDIT 1999-2012**

**REFERENCES**

Auton, A., Abecasis, G.R., Altshuler, D.M., Durbin, R.M., Abecasis, G.R., Bentley, D.R., et al. (2015). A global reference for human genetic variation. *Nature* 526(7571)**,** 68-74. doi: 10.1038/nature15393.

Das, S., Forer, L., Schönherr, S., Sidore, C., Locke, A.E., Kwong, A., et al. (2016). Next-generation genotype imputation service and methods. *Nature Genetics* 48(10)**,** 1284-1287. doi: 10.1038/ng.3656.

Evers, S.E., and Hooper, M.D. (1995). Dietary intake and anthropometric status of 7 to 9 year old children in economically disadvantaged communities in Ontario. *J Am Coll Nutr* 14(6)**,** 595-603. doi: 10.1080/07315724.1995.10718548.

Kim, Y.K., Kim, Y., Hwang, M.Y., Shimokawa, K., Won, S., Kato, N., et al. (2014). Identification of a genetic variant at 2q12.1 associated with blood pressure in East Asians by genome-wide scan including gene-environment interactions. *BMC medical genetics* 15**,** 65-65. doi: 10.1186/1471-2350-15-65.

Solt, F., and Hu, Y. (2019). "interplot: Plot the Effects of Variables in Interaction Terms". package version 0.2.2 ed.).

Xi, B., Zhao, X., Chandak, G.R., Shen, Y., Cheng, H., Hou, D., et al. (2013). Influence of Obesity on Association Between Genetic Variants Identified by Genome-Wide Association Studies and Hypertension Risk in Chinese Children. *American Journal of Hypertension* 26(8)**,** 990-996. doi: 10.1093/ajh/hpt046.
